# Self-organising human gonads generated by a Matrigel-based gradient system

**DOI:** 10.1186/s12915-021-01149-3

**Published:** 2021-09-23

**Authors:** Elizabeth Oliver, João Pedro Alves-Lopes, Femke Harteveld, Rod T. Mitchell, Elisabet Åkesson, Olle Söder, Jan-Bernd Stukenborg

**Affiliations:** 1 NORDFERTIL Research Lab Stockholm, Childhood Cancer Research Unit, J9:30, Department of Women’s and Children’s Health, Karolinska Institutet and Karolinska University Hospital, Visionsgatan 4, 17164 Solna, Stockholm, Sweden; 2 Present address: Wellcome Trust/Cancer Research UK Gurdon Institute, Henry Wellcome Building of Cancer and Developmental Biology, Tennis Court Road, Cambridge, CB2 1QN UK; 3 MRC Centre for Reproductive Health, The Queen’s Medical Research Institute, The University of Edinburgh, 47 Little France Crescent, Edinburgh, Scotland EH16 4TJ UK; 4 Royal Hospital for Children and Young People, 9 Sciennes Road, Edinburgh, EH9 1LF Scotland, UK; 5 Department of Neurobiology, Care Sciences & Society, Division of Neurogeriatrics, Karolinska Institutet, Stockholm, Sweden; 6 The R&D Unit, Stockholms Sjukhem, Stockholm, Sweden

**Keywords:** Testis, Ovary, Organoids, Development, Germ cells, Leydig cells, Sertoli cells

## Abstract

**Background:**

Advances in three-dimensional culture technologies have led to progression in systems used to model the gonadal microenvironment in vitro. Despite demonstrating basic functionality, tissue organisation is often limited. We have previously detailed a three-dimensional culture model termed the three-layer gradient system to generate rat testicular organoids in vitro. Here we extend the model to human first-trimester embryonic gonadal tissue.

**Results:**

Testicular cell suspensions reorganised into testis-like organoids with distinct seminiferous-like cords situated within an interstitial environment after 7 days. In contrast, tissue reorganisation failed to occur when mesonephros, which promotes testicular development in vivo, was included in the tissue digest. Organoids generated from dissociated female gonad cell suspensions formed loosely organised cords after 7 days. In addition to displaying testis-specific architecture, testis-like organoids demonstrated evidence of somatic cell differentiation. Within the 3-LGS, we observed the onset of AMH expression in the cytoplasm of SOX9-positive Sertoli cells within reorganised testicular cords. Leydig cell differentiation and onset of steroidogenic capacity was also revealed in the 3-LGS through the expression of key steroidogenic enzymes StAR and CYP17A1 within the interstitial compartment. While the 3-LGS generates a somatic cell environment capable of supporting germ cell survival in ovarian organoids germ cell loss was observed in testicular organoids.

**Conclusion:**

The 3-LGS can be used to generate organised whole gonadal organoids within 7 days. The 3-LGS brings a new opportunity to explore gonadal organogenesis and contributes to the development of more complex in vitro models in the field of developmental and regenerative medicine.

**Supplementary Information:**

The online version contains supplementary material available at 10.1186/s12915-021-01149-3.

## Background

Human gonad development begins between 3.5 and 4.5 weeks post conception (wpc) during which the primordial germ cells (PGCs) migrate to the primordial gonadal ridge [1]. Sex determination of the bipotential gonad begins at approximately 6 wpc. Somatic cell expression of *SRY* drives human testis development, directing Sertoli cell fate via upregulation of various factors including SOX9 while suppressing genes responsible for ovarian differentiation [1–3].

At the same time as Sertoli cell fate is established, organisation of the seminiferous cords is initiated. Pre-Sertoli cells aggregate and enclose the PGCs (at this stage now termed gonocytes) in partnership with the peritubular myoid precursor cells which encircle the Sertoli-germ cell aggregates leading to clearly visible cords from 7 to 8 wpc [4]. During the process of cord formation, the Sertoli cells begin to express anti-Müllerian hormone (AMH) [4–6], which leads to the regression of the Müllerian structures. Gonocytes in the human embryonic testis are characterised by the expression of pluripotency-associated factors (e.g. POU5F1) which are gradually downregulated from approximately 12 wpc with a reciprocal upregulation of proteins associated with germ cell progression (e.g. MAGE-A4) marking the transition from gonocyte to pre-spermatogonium [4, 7, 8]. Extracellular matrix (ECM) proteins, including fibronectin, collagens, and laminins, are established from week 5 [9]. Collagen 4 and fibronectin can be observed as a net-like arrangement within the interstitium, later forming the basement membrane of the seminiferous tubules, from 6 wpc [9]. The interstitial foetal Leydig cells are established at week 8 facilitating the start of steroidogenesis via the upregulation of genes encoding steroidogenic enzymes including steroidogenic acute regulatory protein (StAR), cytochrome P450 (CYP)17A1, CYP11A1, and hydroxysteroid 17-beta dehydrogenase 3 (HSD17β3) [10]. The ensuing testosterone production is necessary for the maintenance of the Wolffian ducts and their subsequent development into the male reproductive organs (e.g. epididymis, seminal vesicles, and vas deferens). The vascular endothelial cells (VECs) are another interstitial cell population crucial for establishing tissue architecture during testis development. VECs are first observed in the human testis from week 5 with the onset of vessel formation apparent from 7 wpc [4]. Adjacent to the developing gonadal ridge, the mesonephros develops into the Wolffian duct, generating various external urogenital structures as well as contributing endothelial cells to the developing testis [11].

In comparison to the rapid early organisation observed in the testis, morphological changes in the ovary are not apparent until mid-gestation, at which stage follicle formation begins [12]. In the absence of *SRY* expression, initiation of the WNT4/RSPO1/B-catenin signalling pathway in female somatic cells drives granulosa cell differentiation and, in turn, ovarian differentiation. From 6 wpc, pre-granulosa cells expressing ovarian differentiation marker FOXL2 surround clusters of proliferating PGCs (now termed oogonia) forming loosely organised cords and sheets [12–14]. Oogonia continue mitotic proliferation with the gradual onset of meiosis at approximately 14 wpc eventually leading to a loss of pluripotency-associated markers such as POU5F1 [15, 16]. Invasion of germ cell clusters by pre-granulosa cells from 14 wpc leads to the formation of individual primordial follicles [17]. Early embryonic development in both sexes is therefore crucial for establishment of the somatic cell environment which will support germ cell development during adulthood, laying down the foundations for subsequent reproductive success [18].

Numerous culture approaches have been employed to recapitulate and investigate the gonadal microenvironment in vitro [19, 20]. Dissociated testicular cells can be integrated into various matrix scaffolds including soft agar [21, 22], collagen gel [23], Matrigel [24], or decellularised testicular matrix [25–27] to generate three-dimensional (3D) structures in order to examine the gonadal microenvironment in vitro. Alternatively, cells can reorganise without the need of a scaffold, forming multi-cellular aggregates using methods such as the hanging drop system [28, 29], microwell aggregation [30, 31], or suspension-based culture [32]. Despite comprising key cell types and demonstrating basic functionality, tissue organisation in current approaches generally consists of multi-cellular aggregates forming individual tubule-like structures [24, 28, 30]. This contrasts a whole testis structure with structurally discrete compartments and conserved paracrine signalling necessary for maturation of the somatic cell niche and germ cell differentiation.

Here we describe the generation of novel self-organising compartmentalised human gonadal organoids. The approach is an extension of our previously detailed 3D testicular model termed the three-layer gradient system (3-LGS) used to generate rat testicular organoids in vitro [33, 34]. Using a multilayer approach, in which dissociated testicular tissue was embedded in a layer of Matrigel situated between two cell free layers of the same matrix, we describe the reorganisation of tubule-like structures situated within an interstitial environment separated by a basement membrane.

## Results

### The 3-LGS generates organoids with compartmentalised testis-like architecture

**Fig. 1:**
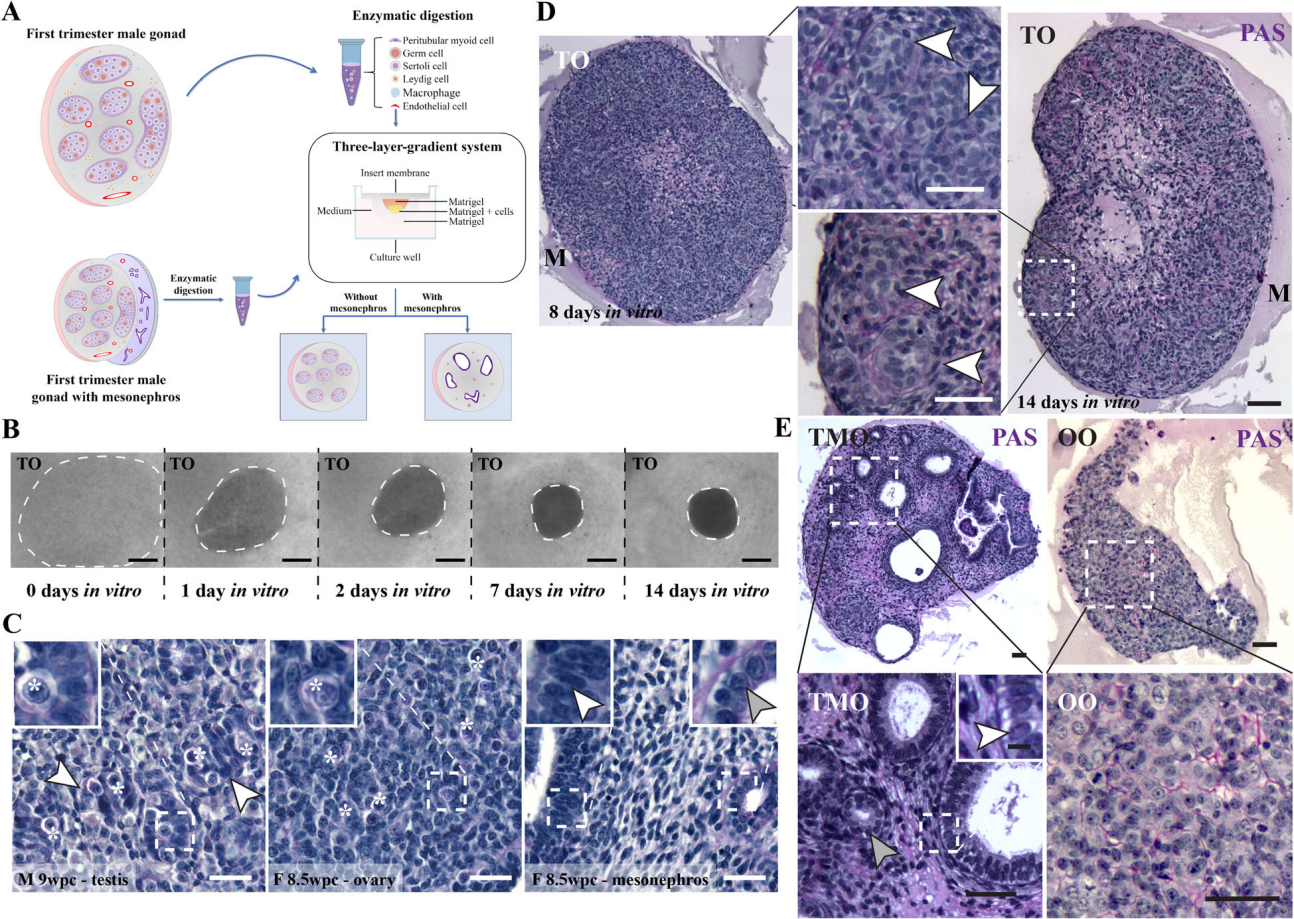
The 3-LGS generates compartmentalised human gonadal organoids. **A** Schematic illustrating culture preparation and the 3-LGS setup. **B** Organoid formation in culture following testicular tissue dissociation. Images illustrate the re-organisation of gonad tissue from small cell aggregates and single cells from the same 8.5–9 wpc embryonic tissue sample. Scale bars, 500 μm. **C** Representative images from 8.5 wpc female and 9 wpc male embryonic gonads (PAS, periodic acid-Schiff). Female gonad accompanied by mesonephric tissue observed as small tubular lumens lined by a simple cuboidal epithelium (grey arrow, F 8.5 wpc—mesonephros inset) and larger ducts with a pseudostratified columnar epithelium (white arrow, F 8.5 wpc—mesonephros inset). Germ cells (asterisks) situated throughout the ovary, whereas localised within seminiferous cords (as indicated by white arrow, M 9 wpc zoom) in the testis. Scale bars, 50 μm. **D** Dissociated testicular cells reorganise into testicular organoids (TO) with distinct seminiferous-like cords (white arrows) situated within an interstitial environment after seven days in the 3-LGS and maintain structure until the end of the 14-day culture period (representative organoid images from the same 8.5–9 wpc embryonic tissue sample) (PAS, periodic acid-Schiff). **E** Tissue reorganisation fails to occur when mesonephros is included in the testicular tissue digest (testicular mesonephric organoid (TMO); representative organoid image from a 7.5 wpc embryonic tissue sample). The inclusion of mesonephric tissue resulted in the formation of mesonephric-like tubules throughout the interstitial space, observed as small tubular lumens lined by a simple cuboidal epithelium (grey arrow) or larger ducts with a pseudostratified columnar epithelium (white arrow; inset). The 3-LGS can also be used to generate ovarian organoids (OO) from dissociated ovarian tissue (representative organoid image from a 10 wpc embryonic tissue sample)

To generate organoids, we cultured dissociated first-trimester embryonic tissue from the gonadal ridge using the 3-LGS for 7 to 14 days (Fig. 1A). In the embryonic testis, seminiferous tubule formation is initiated by 6 wpc with cords clearly visible by 7 to 8 weeks [4]. Accordingly, dissociated testis tissue comprising small cell aggregates and single cells (*n* = 3; 8, 8.5, and 8.5–9 wpc) reaggregated (Fig. 1B) and reorganised into testis-like organoids (TO) with distinct seminiferous-like cords situated within an interstitial environment similar to morphological structures observed in age-matched controls within 7 days (Fig. 1C, D). The 3-LGS maintained the compartmentalised organoid structure until the end of the 14-day culture period. In contrast, tissue reorganisation failed to occur in two out of three samples (*n* = 3; 5–6, 7.5, 9.5 wpc) when mesonephros, which promotes testicular development in vivo, was included in the tissue digest forming testicular mesonephric organoids (TMO) (Fig. 1E) [11]. The inclusion of mesonephric tissue resulted in the formation of mesonephric-like tubules throughout the interstitial space. These were observed as small tubular lumens lined by a simple cuboidal epithelium or larger ducts with a pseudostratified columnar epithelium (Fig. 1E). Ovarian organoids (OO) generated from dissociated female gonad cell suspensions (*n* = 3; 5.5, 9.5, 10 wpc) formed loosely organised cords after 7 days (Fig. 1E), typical of the more limited organisation observed (when compared to the testis) from 6 wpc until mid-gestation as illustrated in control tissue (Fig. 1C) [12].

**Fig. 2:**
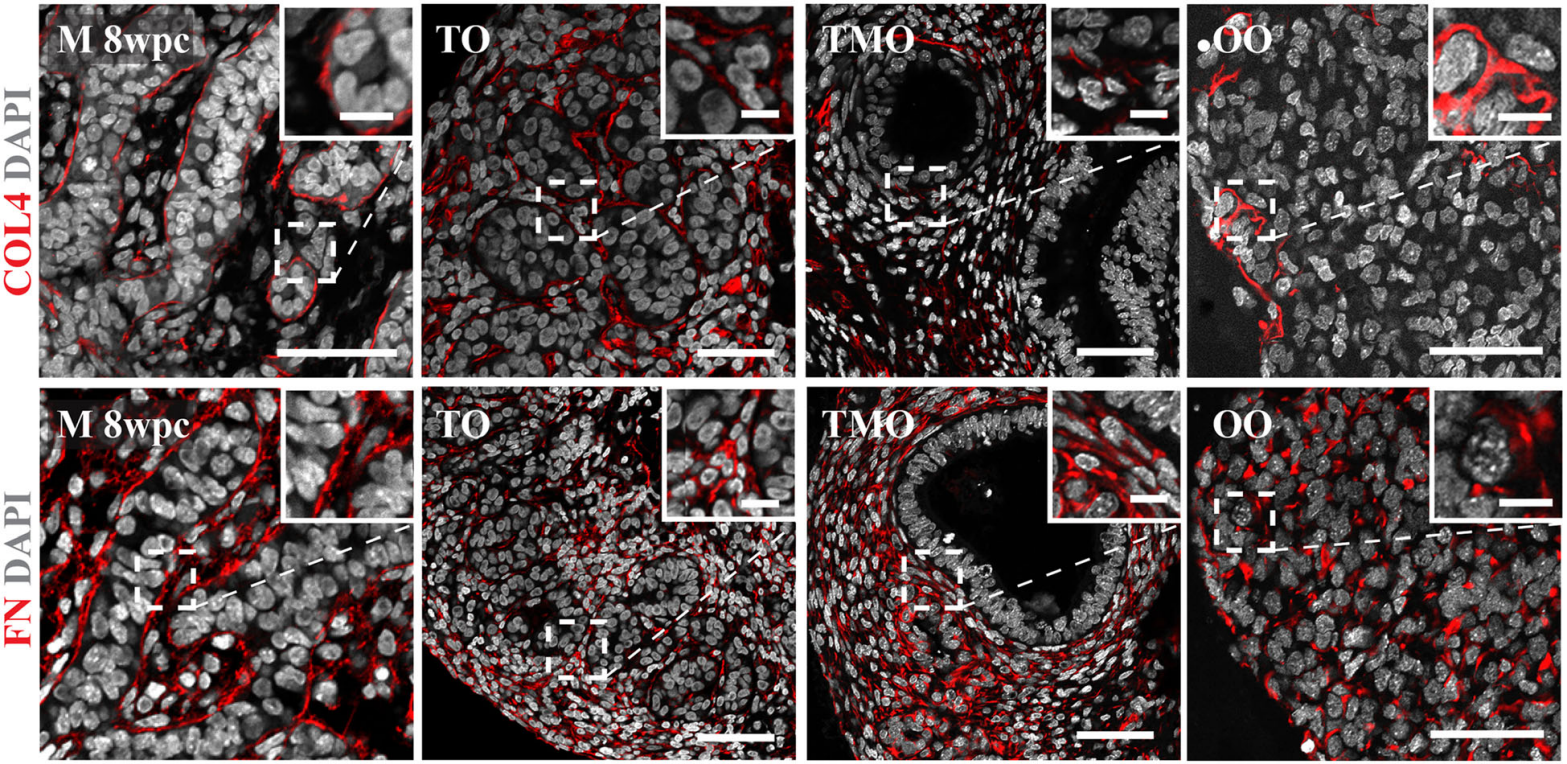
Expression of basement membrane proteins in the 3-LGS. Immunolabelling of basement membrane proteins collagen 4 (COL4) and fibronectin (FN) (both red) highlight the compartmentalised testicular organoid structure (TO) (representative organoid image from 8 wpc embryonic tissue sample) and the lack of organisation observed in testicular mesonephric organoids (TMO) (representative organoid image from 7.5 wpc embryonic tissue sample) when compared to male (M) 8 wpc in vivo control. Collagen 4 immunostaining revealed the onset of early tissue organisation in ovarian organoids (OO) (representative organoid image from 10 wpc embryonic tissue sample). All organoid images from day 14 culture samples unless otherwise stated. Scale bars, 50 μm (insets, 10 μm)

Collagen 4 and fibronectin were observed in the establishing basement membrane revealing a compartmentalised testis-like architecture (Fig. 2). When generated in the presence of mesonephros, organoids displayed a largely ubiquitous expression of collagen 4 and fibronectin confirming an absence of seminiferous-like cord formation (Fig. 2). Fibronectin expression was widespread in the ovarian organoids (Fig. 2). In contrast, collagen 4 fibrils were more limited, but revealed the onset of early tissue organisation as described in vivo [35].

### The 3-LGS supports the somatic cell environment

**Fig. 3:**
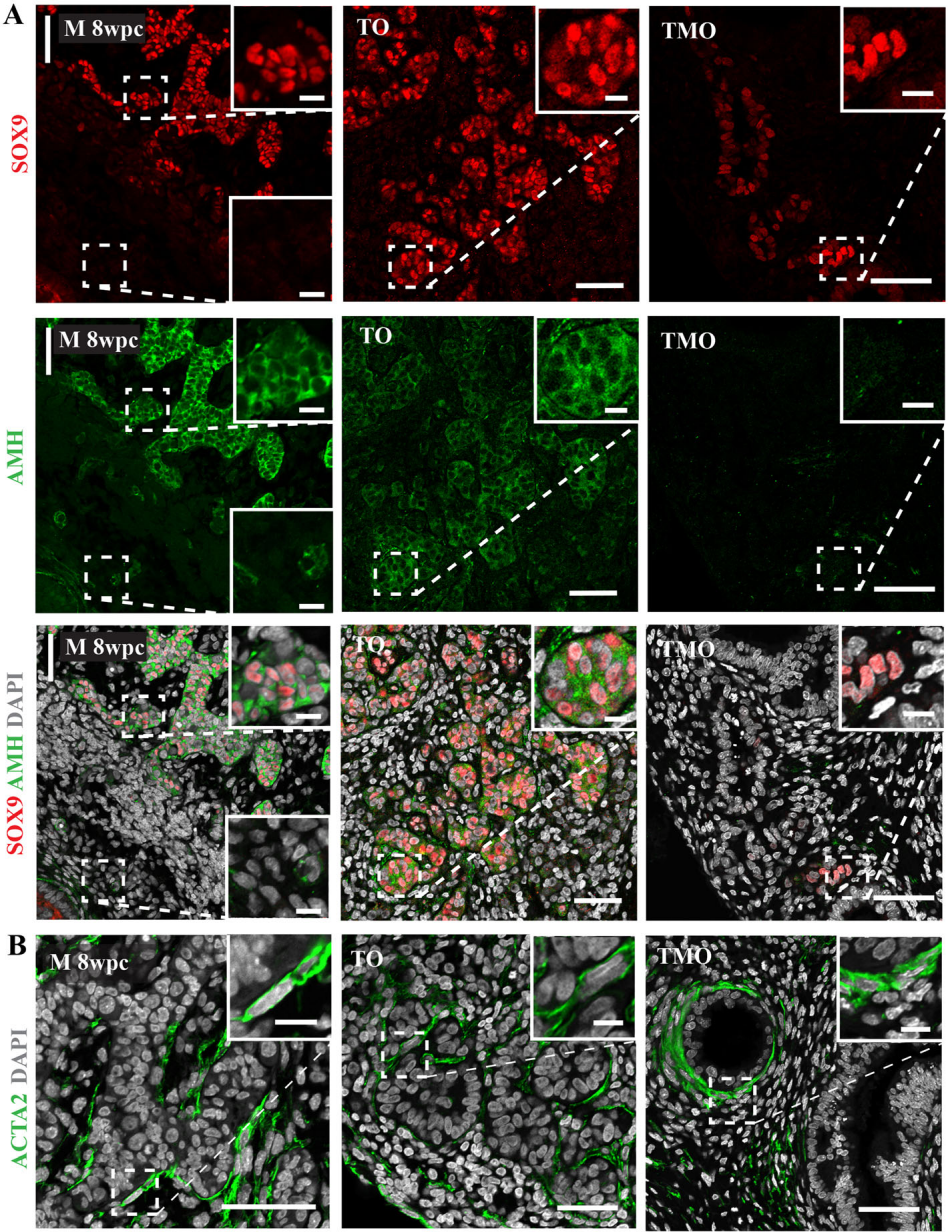
The 3-LGS supports the male somatic cell environment. **A** Immunolabelling of Sertoli cell marker SOX9 (red) and cytoplasmic anti-Müllerian hormone (AMH) (green) within newly formed cords in testicular organoid (TO) (representative organoid image from 8 wpc embryonic tissue sample). **B** Actin alpha 2 (ACTA2) (green) expressing vascular smooth muscle cells and peritubular myoid cells in TO (representative organoid image from 8 wpc embryonic tissue sample) and testicular mesonephric organoid (TMO) (representative organoid image from 7.5 wpc embryonic tissue sample). All organoid images from day 14 culture samples. In vivo control male (M) 8 wpc testis. Scale bars, 50 μm (insets, 10 μm)

During the process of cord formation, Sertoli cells begin to express AMH, a characteristic of Sertoli cell differentiation [4–6]. In the 3-LGS, we observed Sertoli cell marker SOX9 within newly formed cords accompanied by cytoplasmic AMH expression, suggesting that organoids can support this aspect of somatic cell function (Fig. 3A and Additional file 1: Fig. S1A). In contrast, no AMH was detected in SOX9-positive cells in TMO suggesting a lack of Sertoli cell differentiation (Fig. 3A). Actin alpha 2 (ACTA2)-positive peritubular myoid cells were distinguishable around the newly organised seminiferous cords as observed in vivo as well as surrounding the mesonephric-like tubules in TMOs (Fig. 3B).

To examine levels of apoptosis in the culture system, immunostaining for cleaved caspase-3 was evaluated. Very few apoptotic cells were detected in the cultures (Additional file 2: Fig. S2A) and levels were comparable to those observed in age-matched in vivo tissue suggestive of a viable system for cell survival. Moreover, cell proliferation was demonstrated by Ki67 expression in all organoids (Additional file 2: Fig. S2B). The onset of vasculature formation within the interstitial space was demonstrated at 14 days by CD31-positive endothelial cells; however, this was only observed in one of the TOs (Additional file 2: Fig S2C).

### Expression of steroidogenic pathway enzymes in the 3-LGS

**Fig. 4:**
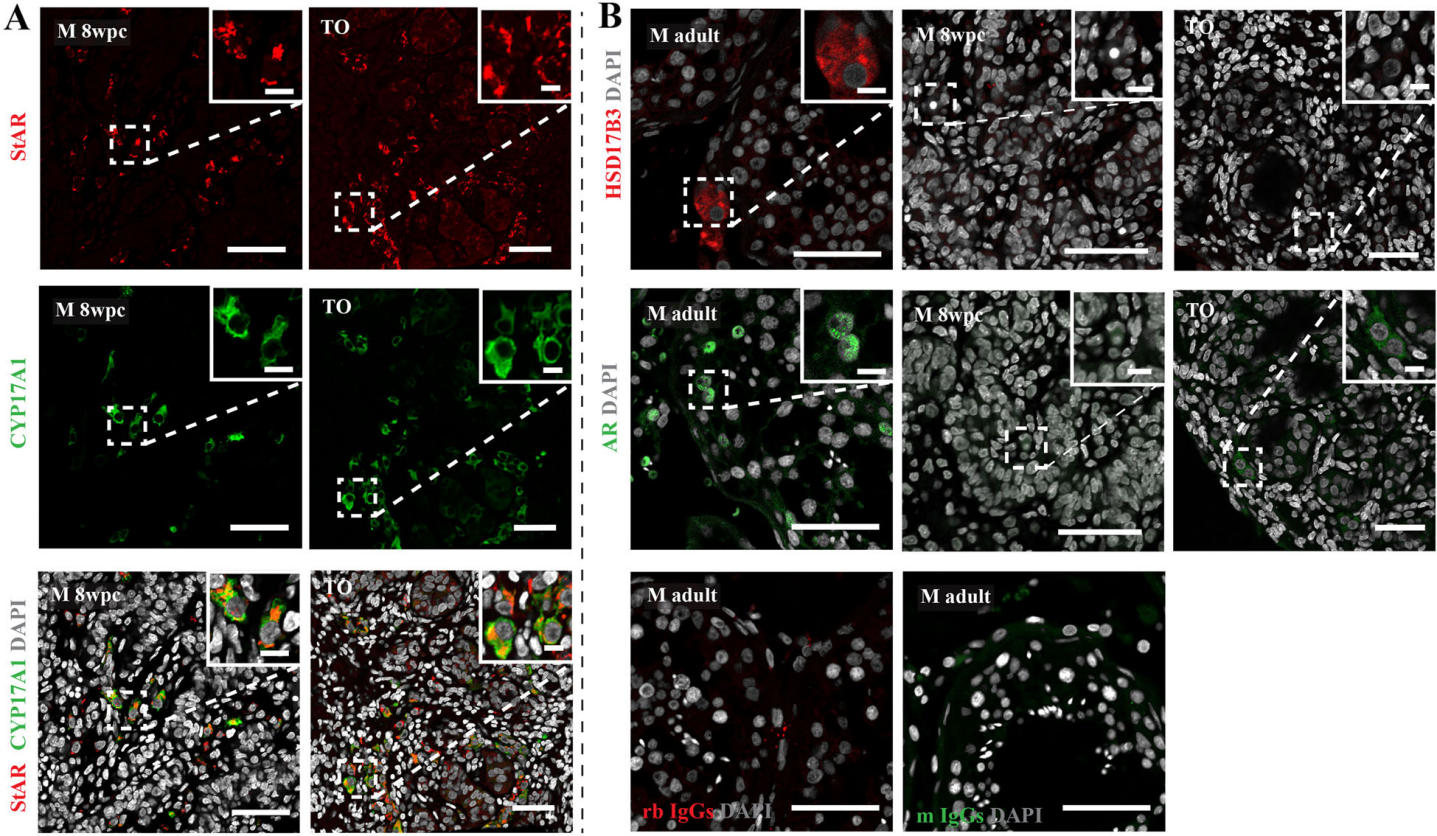
Expression of steroidogenic pathway enzymes in the 3-LGS. **A** Immunolabelling of steroidogenic enzyme markers StAR (red) and CYP17A1 (green) revealed interstitial expression in all testicular organoids (TO) (representative organoid image from 8 wpc embryonic tissue sample). In vivo control male (M) 8 wpc testis. **B** No nuclear androgen receptor (AR) (green) expression in the interstitial compartment (representative image from 8 wpc TO sample) was observed. No 17-HSDβ3 (red) expression was detected. In vivo control male (M) 8 wpc and adult testis. Negative IgG isotype controls (rabbit (rb) (red) and mouse (ms) (green)). All organoid images from day 14 culture samples. Scale bars, 50 μm (insets, 10 μm)

The steroidogenic Leydig cells populate the interstitial compartment of the testis from 8 wpc at which time they begin to synthesise testosterone [10]. To investigate steroidogenic capacity, organoids were examined for markers of the steroidogenic pathway, including StAR, CYP17A1, and 17-HSDβ3. StAR and CYP17A1-positive Leydig cells were observed in the interstitium of all organoids similar to 8 wpc control tissue (Fig. 4A and Additional file 1: Fig. S1A), whereas 17-HSDβ3 staining was not detected in either organoids or age-matched control tissue (Fig. 4B). When quantified, an increasing trend (*p* = 0.09) in the number of CYP17A1-positive cells was observed from day 7 (0.42 ± 0.41 [mean ± SD]%) to day 14 day (2.25 ± 1.70%) (Additional file 1: Fig. S1A). Staining for androgen receptor (AR) revealed neither nuclear expression in the interstitial compartment in TOs nor in 8 wpc control tissue (Fig. 4B).

### Germ cell maintenance in the 3-LGS

**Fig. 5:**
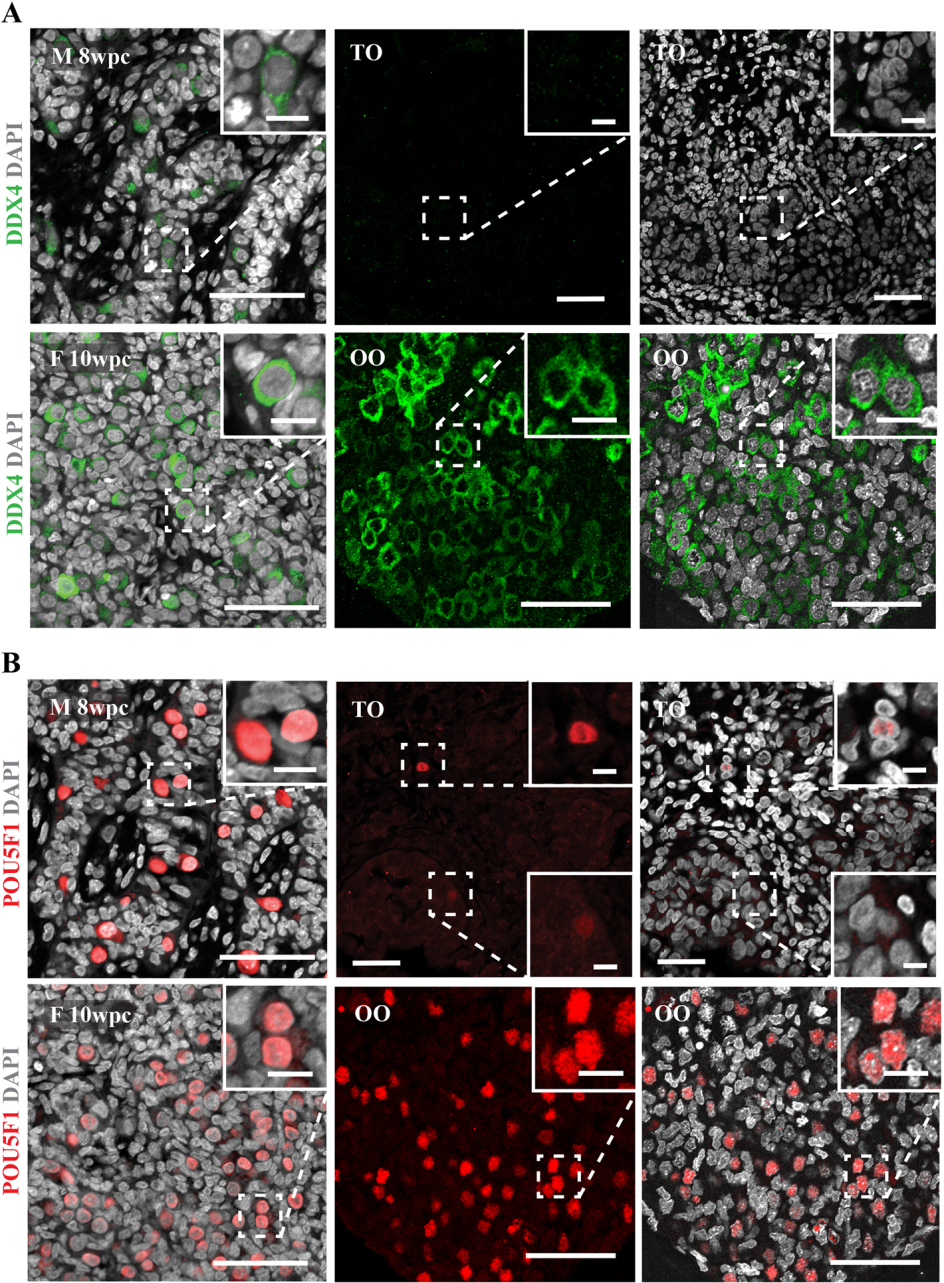
The 3-LGS maintains germ cell survival in the female but not the male. Ovarian organoids (OO) support germ cell survival throughout culture as indicated by immunolabelling for both **A** DDX4 (primordial germ cell marker) (green) and **B** POU5F1 (pluripotency marker) (red) (representative organoid images from 10 wpc embryonic tissue sample). In vivo control female (F) 10 wpc ovary. No DDX4-positive cells were observed in testicular organoids (TO) (**A**); however, a limited number of POU5F1-positive cells (**B**) were detected (representative images from 8 wpc embryonic tissue sample). In vivo male germ cell distribution demonstrated in 8 wpc control. All images from day 14 culture samples. Scale bars, 50 μm (insets, 10 μm)

Following characterisation of the somatic environment, organoids were examined for the presence of germ cells. In the embryonic testis, the transition from gonocytes to pre-spermatogonia occurs from approximately week 12, marked by the downregulation of pluripotency related factors (e.g. POU5F1) [8]. While no DDX4-positive cells were observed in TOs (Fig. 5A), a small number of DAZL and POU5F1-positive cells were detected at both day 7 (DAZL 0.03 ± 0.05%, OCT4 0.35 ± 0.22%) and day 14 (DAZL 0.16%, OCT4 0.06 ± 0.07%), located within tubule-like cords and interstitial space (Fig. 5B and Additional file 1: Fig. S1B). While quantification of POU5F1-positive cells revealed a gradual loss during culture, numbers were already significantly depleted at day 7 when qualitatively compared to age-matched controls (Fig. 5B), suggesting that the vast majority of germ cell loss occurs between tissue dissociation and day 7. In contrast, DDX4- and POU51-positive germ cells appeared comparable to the situation in vivo in organoids generated from dissociated female gonads (Fig. 5A, B).

## Discussion

Studies reporting the generation of human testicular organoids describe 3D cell clusters containing germ cells surrounded by a disorganised aggregation of somatic cells [28] or structures which assume a reverse polarity, whereby a centrally located interstitial compartment is enclosed by a basement membrane and Sertoli cells [30]. Here we report the formation of compartmentalised whole human embryonic testicular organoids comprising cord-like structures situated within an interstitial environment. The organoids are compartmentalised by a basement membrane and maintain key somatic cell types including Sertoli, Leydig and peritubular myoid cells.

In addition to displaying testis specific architecture, organoids demonstrated evidence of somatic cell differentiation. Within the 3-LGS, we observed the onset of AMH expression in the cytoplasm of SOX9-positive Sertoli cells within reorganised testicular cords. Both Sertoli and peritubular myoid cells contribute to the production of basement membrane components, including collagen 4 and fibronectin, which are deposited at the interface between the two cell types [36]. Accordingly, collagen 4 and fibronectin were observed in the establishing basement membrane and interstitial compartment of testicular organoids indicative of peritubular myoid cell function. Leydig cell differentiation and onset of steroidogenic capacity was also revealed in the 3-LGS through the expression of key steroidogenic enzymes StAR and CYP17A1 within the interstitial compartment.

Previous studies suggest that facilitating direct cell-cell interactions such as those achieved through encapsulation of testicular cells within a 3D scaffold (hydrogels or decellularised testis) or cellular aggregation (microwell or suspension-based culture) may be beneficial for cell assembly and self-organisation. In conventional 3D models, dissociated cells are typically distributed equally throughout the culture microenvironment. The 3-LGS expands on this strategy using a multilayer system whereby dissociated testicular tissue is embedded in a layer of Matrigel situated between two cell free layers. We propose that the success of the 3-LGS system centres on the generation of two concentration gradients formed by the layered structure—the inflow of factors from the Matrigel and culture medium (to be consumed by the cells) and the subsequent outflow of cellular metabolites. Supporting this hypothesis, we demonstrated in rats that tubule-like structures do not reorganise in a single layer of Matrigel using the same volume and cell concentration as used in the 3-LGS [33]. A recent study from ME Edmonds and TK Woodruff [37] suggests that Matrigel ECM does not benefit organoid formation in a 3D environment. Substitution of Matrigel with an alternative gel scaffold in the 3-LGS would therefore be informative to determine whether it is the Matrigel constituents or the three-layered structure per se that contributes to the high level of tissue reorganisation observed in our study.

In vivo the mesonephros, comprised of glomeruli and mesonephric tubules, functions as a temporary kidney up to 8 wpc [38]. The mesonephros further promotes testicular development, contributing endothelial cells to the developing testis [11], and following its regression, the remaining mesonephric tubules form the efferent ductules [38]. Despite this, inclusion of mesonephros in the tissue digest impaired the formation of testicular organoid structures. While the reason for this is unclear, we speculate that paracrine factors released by the mesonephric cells block the reorganisation of the gonad tissue. Alternatively, disruption caused by the mesonephric tissue may simply be a result of uncontrolled proliferation and expansion of these cells, leading to perturbation of the comparatively slower reorganising gonad tissue.

A challenge facing the bioengineering of testicular organoids is the survival, maintenance, and differentiation of germ cells. Given that germ cells can be maintained in the female somatic environment within the 3-LGS, it appears that the loss of germ cells observed in the male environment is not an issue intrinsic to the culture system. When and how germ loss occurs in the cultured male microenvironment and not the female is unclear but could be investigated by use of a chimeric approach comprising a female derived somatic environment combined with male germ cells. A possible cause could be a loss of germ cell to Sertoli cell contact leading to increased exposure of germ cells to retinoic acid, resulting in premature differentiation and, consequently, apoptosis. Alternatively, germ cell apoptosis may result from activation of the innate immune response in culture as recently described in a mouse testis organ culture [39]. Use of the 3-LGS would offer a robust approach by which to address these and other future research questions.

A growing body of evidence suggests that male reproductive disorders, such as decreased sperm count and increased risk of testicular cancer, originate during prenatal development [40]. Based on timings in rats, the developmental window at risk of disruption in humans is thought to be approximately 8–14 weeks of gestation [41]. While the underlying cause remains unclear, maternal exposure to environmental endocrine-disrupting chemicals has been suggested as one such contributory factor. A suitable model system of in vitro gonadal development would enable a better understanding of such disorders and their origins. The 3-LGS can be used to generate organised whole gonadal organoids after 7 days. In contrast to models which make use of intact tissue pieces, the 3-LGS can be used to track various cell populations and their interactions during development, examine the impact of exogenous factors on organogenesis as well as allowing for easy manipulation of cell populations through their inclusion or exclusion. The method may also support the formation of organoids from pluripotent stem cells or primary cells from other human tissues of interest, providing additional model systems for regenerative medicine.

## Conclusion

We conclude that the 3-LGS can be used to generate organised gonadal organoids within 7 days. Moreover, the exclusion of the in vivo supporting mesonephric tissue improves the reorganisation of the dissociated gonadal tissue in vitro. Additionally, the maintenance of germ cells in the organoids after the dissociation and the reorganisation processes is gender dependent: germ cells can be maintained in the ovarian but not in the testicular organoids. The 3-LGS brings a new opportunity to explore gonadal organogenesis and contributes to the development of more complex in vitro models in the field of developmental and regenerative medicine.

## Methods

### Human tissue collection

First-trimester human embryonic gonads between 5 and 9.5 wpc were collected following elective termination of pregnancy and either processed for culture or fixed in 4% paraformaldehyde. Tissue staging (accuracy ± 0.5 weeks) was determined by examination of anatomical landmarks in addition to clinical ultrasound. Sex determination was established using embryonic control tissue. Genomic DNA was extracted using the DNA Mini kit (Qiagen, 51306) according to the manufacturer’s instructions. In brief, tissue was lysed using Proteinase K at 56 °C. DNA was adsorbed onto silica membranes and transferred to spin columns. Following a series of washing steps, purified DNA was eluted. PCR was performed using primers for SRY (primer sequence (5′ to 3′) forward: TCG CGA TCA GAG GCG CAA GA; reverse: GCT GCG TTG ATG GGC GGT AA; reverse: GCT GCG TTG ATG GGC GGT AA) and β-actin as an internal reference control gene (primer sequence (5′ to 3′)): forward: CAT GTA CGT TGC TAT CCA GGC; reverse: CTC CTT AAT GTC ACG CAC GAT) followed by agarose gel electrophoresis. Adult control testis from non-diseased patients were obtained from the University of Edinburgh and fixed in 10% Neutral-buffered formalin and paraffin embedded.

### Tissue dissociation

Embryonic gonads with or without mesonephros (random allocation) were mechanically fragmented into approximately 0.5 mm^3^ pieces using a scalpel and enzymatically digested in Minimum essential medium alpha (MEM-α) (Gibco, 22561-021) supplemented with 1 mg/ml collagenase 1A (Sigma-Aldrich, C2674), 0.5 mg/mL DNase type 1 (Roche Diagnostics, 10104159001), and 0.5 mg/ml hyaluronidase (Sigma-Aldrich, H3506) for 15 min, at 37 °C and 120 rpm. Small cell aggregates and single cells were sedimented for 5 min at 300×*g* and 4 °C and resuspended in NutriStem (Biological Industries, 05-100-1A) supplemented with 1% Penicillin/Streptomycin (Gibco, 15070-063). Cell viability and concentration were determined.

### 3-LGS culture

Dissociated embryonic gonad tissue was cultured in a 3-LGS as previously described [34]. Briefly, dissociated cells and small aggregates were suspended in Matrigel (Corning, 356231) diluted 1:1 in MEM-α, 1% (vol/vol) Penicillin/Streptomycin at a concentration of 44, 000, 000 cells per ml (132, 000 cells in 3 μl). Three concentric drops of Matrigel (5 μl Matrigel, 3 μl Matrigel-cell suspension, 8 μl Matrigel) were sequentially plated onto the underside cell membrane of a hanging cell insert and allowed to solidify. Hanging cell inserts were positioned in 24-well plates (VWR, 734-2325) and suspended in 600 μl of NutriStem supplemented with 10% KnockOut serum replacement (Gibco, 10828-028) and 1% Penicillin/Streptomycin. Organoids were maintained under humidified conditions at 37 °C (5% CO_2_) for up to 14 days and 300 μl medium was replaced every second day.

### Immunofluorescence

Following culture testicular organoids were fixed in 4% paraformaldehyde (HistoLab, 02176) or Bouin’s solution (Sigma-Aldrich, HT10132) and processed for immunofluorescence. Briefly, organoids were embedded in paraffin and sectioned (5 μm). Sections were dewaxed and rehydrated before boiling in Tris-EDTA buffer (10 mM, pH 9) with 0.05% Tween 20. To reduce non-specific binding, sections were blocked with 10% normal donkey serum (Jackson ImmunoResearch, 017-000-121) and 4% bovine serum albumin (Sigma-Aldrich, A2153) for one hour at room temperature. Primary antibodies (listed in Table 1) were diluted in blocking buffer and sections incubated overnight at 4 °C. Corresponding species-specific IgG isotype controls (normal rabbit IgG (Abcam ab27478); normal mouse IgG (Santa Cruz Biotechnology sc-2025)) were included in each experiment at concentration equivalent to the primary antibody (Fig. S1D). Sections were incubated with secondary antibodies (1:500, donkey anti-rabbit Cy3 (ThermoFisher 11483299) or donkey anti-mouse AlexaFluor® 488 (Thermo Fisher 715546150)) for 60 min before mounting with Prolong® Gold anti-fade reagent with DAPI (Invitrogen, P36931). Images were recorded with a Leica inverted SP-5 confocal microscope. Additional sections were stained for periodic acid-Schiff according to manufacturer’s instructions (Merck, 101646) and counter-stained with haematoxylin (Merck, 1092491000).

**Table 1:** List of antibodies

Antibody	Species	Concentration (mg/ml)	Catalogue number	Resource Identification Portal
ACTA2	Mouse	1:500 dilution	A2547	Sigma-Aldrich Cat# A2547, RRID:AB_476701
AMH	Mouse	0.004	sc-166752	Santa Cruz Biotechnology Cat# sc-166752, RRID:AB_2289536
AR	Mouse	0.004	sc-7305	Santa Cruz Biotechnology Cat# sc-7305, RRID:AB_626671
CD31	Mouse	0.05	ab119339	Abcam Cat# ab119339, RRID:AB_10936456
COL IV	Rabbit	0.001	ab214417	Abcam Cat# ab214417, RRID:AB_2801511
CYP17A1	Mouse	0.001	sc-374244	Santa Cruz Biotechnology Cat# sc-374244, RRID:AB_10988393
DDX4	Mouse	0.01	ab27591	Abcam Cat# ab27591, RRID:AB_11139638
FN	Rabbit	0.008	ab32419	Abcam Cat# ab32419, RRID:AB_732379
POU5F1	Rabbit	0.005	ab19857	Abcam Cat# ab19857, RRID:AB_445175
SOX9	Rabbit	0.005	AB5535	Millipore Cat# AB5535, RRID:AB_2239761
StAR	Rabbit	0.004	sc-25806	Santa Cruz Biotechnology Cat# sc-25806, RRID:AB_2115937
17-HSDβ3	Rabbit	0.004	sc-135043	Santa Cruz Biotechnology Cat# sc-135043, RRID:AB_10709740
DAZL	Rabbit	0.003	ab215718	Abcam Cat# ab215718, RRID:AB_2893177
CASP3	Rabbit	1:100 dilution	9661	Cell Signaling Technology Cat# 9661, RRID:AB_2341188.

*ACTA2* actin alpha 2, *AMH* anti-Müllerian hormone, *AR* androgen receptor, *FN* fibronectin

Immunohistochemical quantification of germ cell numbers (POU5F1 and DAZL) in the 3-LGS was performed on central sections of testicular organoids at culture day 7 and 14. CYP17A1-positive cells were similarly quantified to confirm active protein turnover and cell differentiation in the 3-LGS. For each organoid analysed, the number of somatic cell nuclei positive for DAPI in one or two central sections was counted, as were those positive for the marker of interest (POU5F1, DAZL, or CYP17A1). The proportion of positive cells relative to the total cell count was calculated for each section and data presented as mean ± SD. Analysis was performed using Microsoft Excel and data were analysed by unpaired *t*-test with statistical significance considered to be *p <* 0.05.

## Supplementary Information

**Additional file 1: Fig. S1. A** Immunolabelling of Sertoli cell marker SOX9 (red), cytoplasmic anti-Müllerian hormone (AMH) (green) and steroidogenic enzyme marker CYP17A1 (green) in testicular organoids (TO) at culture day 7 and day 14 (representative organoid image from 8 wpc embryonic tissue sample). **B** A limited number of POU5F1 and DAZL-positive cells were detected at day 7 suggesting that the vast majority of germ cell loss occurs between digestion and day 7 (representative organoid image from 8.5-9 wpc embryonic tissue sample). Scale bars, 50 μm (insets, 10 μm).

**Additional file 2: Fig. S2. A** Immunolabelling of apoptosis marker Caspase-3 (CASP3) and **B** proliferation marker KI67 (both red) in testicular organoid (TO), testicular mesonephric organoid (TMO) and ovarian organoid (OO) (representative organoid images from 8, 7.5 and 10 wpc embryonic tissue samples respectively). In vivo control male (M) 8 wpc testis, male mesonephros (MM) 8 wpc and female (F) 10 wpc ovary. **C** CD31 expressing endothelial cells (green) were observed in the interstitium of one TO (representative image from 8 wpc embryonic tissue sample) but not detected in any of the TMOs. **D** Negative controls rabbit (rb) (green) and mouse (m) (red) IgG (control tissue 8 wpc testis). All images from day 14 culture samples. Scale bars, 50 μm (insets, 10 μm).

## Data Availability

All data generated or analysed during this study are included in this published article and its supplementary information files. The datasets used and/or analysed during the current study are also available from the corresponding author on request.
