# Supplementary figures and images for: Self-organising human gonads generated by a Matrigel-based gradient system
Source: BMC Biol. 2021 Sep 23;19:212. doi: 10.1186/s12915-021-01149-3 (PMC8461962; doi:10.1186/s12915-021-01149-3)

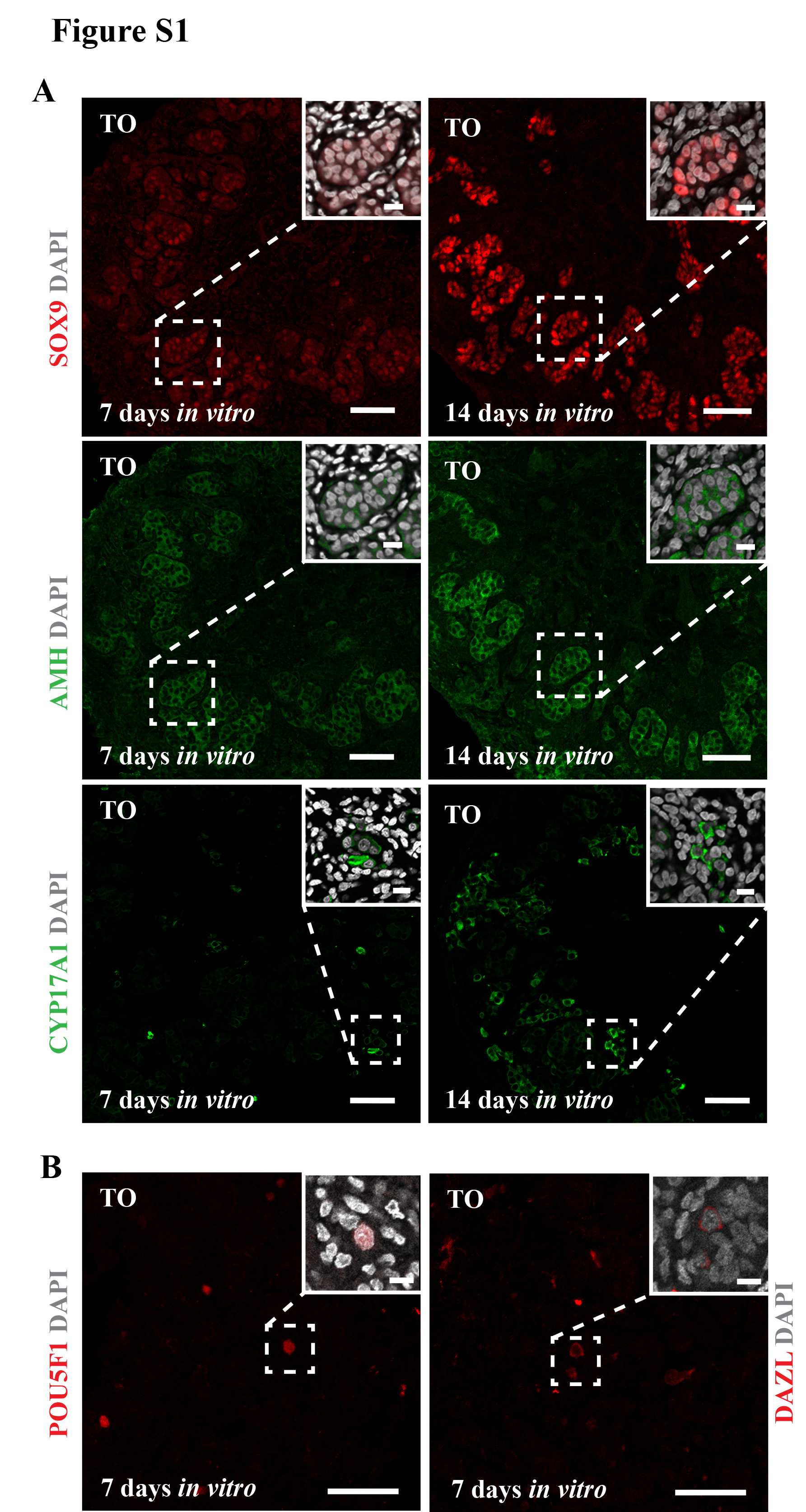

Supplement: Supplementary file 1 — Additional file 1: Fig. S1. A Immunolabelling of Sertoli cell marker SOX9 (red), cytoplasmic anti-Müllerian hormone (AMH) (green) and steroidogenic enzyme marker CYP17A1 (green) in testicular organoids (TO) at culture day 7 and day 14 (representative organoid image from 8 wpc embryonic tissue sample). B A limited number of POU5F1 and DAZL-positive cells were detected at day 7 suggesting that the vast majority of germ cell loss occurs between digestion and day 7 (representative organoid image from 8.5-9 wpc embryonic tissue sample). Scale bars, 50 μm (insets, 10 μm). [file 12915_2021_1149_MOESM1_ESM.jpg]

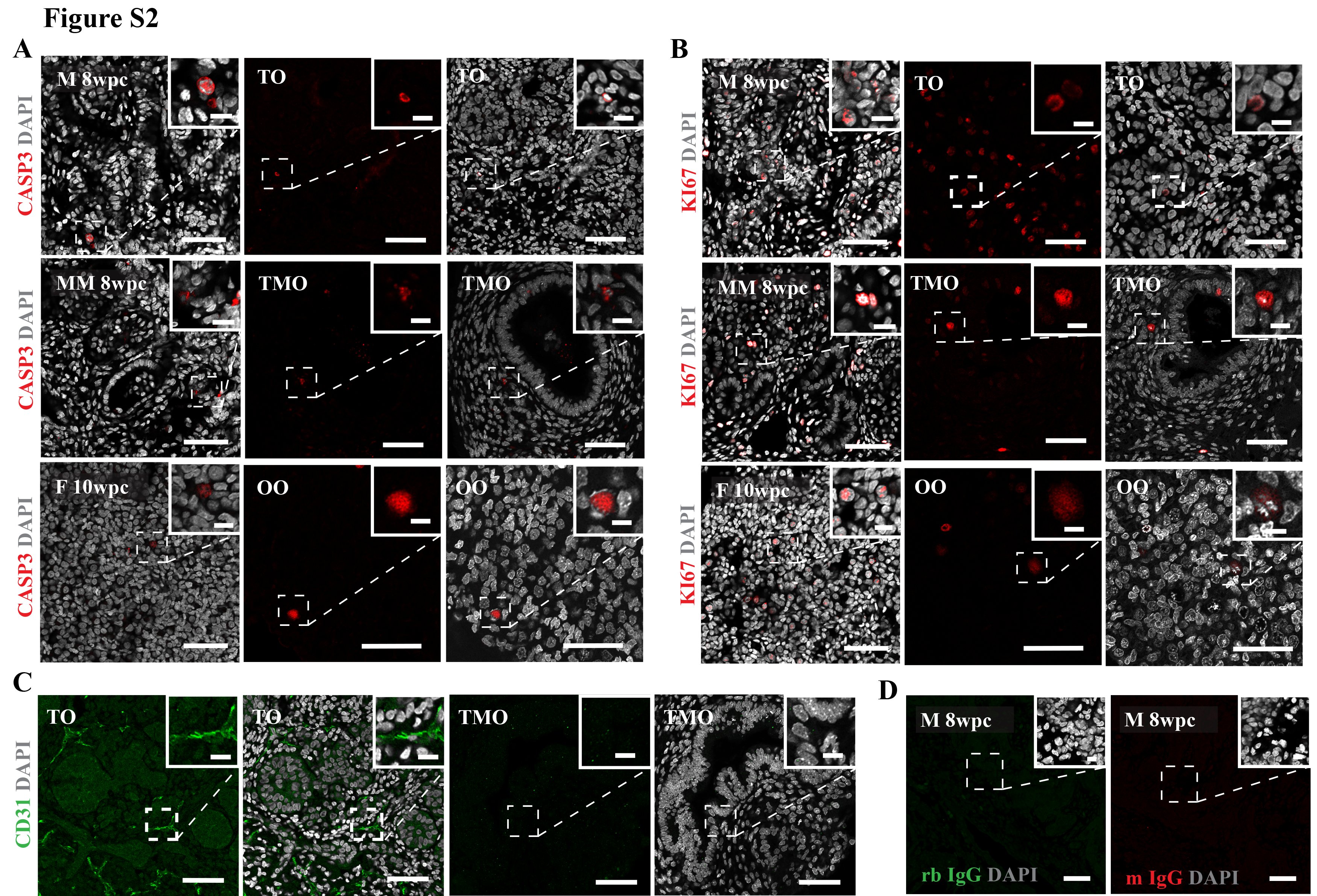

Supplement: Supplementary file 2 — Additional file 2: Fig. S2. A Immunolabelling of apoptosis marker Caspase-3 (CASP3) and B proliferation marker KI67 (both red) in testicular organoid (TO), testicular mesonephric organoid (TMO) and ovarian organoid (OO) (representative organoid images from 8, 7.5 and 10 wpc embryonic tissue samples respectively). In vivo control male (M) 8 wpc testis, male mesonephros (MM) 8 wpc and female (F) 10 wpc ovary. C CD31 expressing endothelial cells (green) were observed in the interstitium of one TO (representative image from 8 wpc embryonic tissue sample) but not detected in any of the TMOs. D Negative controls rabbit (rb) (green) and mouse (m) (red) IgG (control tissue 8 wpc testis). All images from day 14 culture samples. Scale bars, 50 μm (insets, 10 μm). [file 12915_2021_1149_MOESM2_ESM.jpg]
